# Prognostic factors and nomogram-based survival prediction for patients with terminal-stage cancer: A retrospective study

**DOI:** 10.1097/MD.0000000000045262

**Published:** 2025-10-17

**Authors:** Weiwei Gui, Chunhui Ding, Lei Xu, Yizhou Luo, Lingxiang Liu

**Affiliations:** a Department of Oncology of Hospital, Nanjing Tech University, Nanjing, China; b Department of Internal Medicine of Hospital, Nanjing Tech University, Nanjing, China; c Department of Oncology, Air Force Hospital of the People’s Liberation Army Eastern Theater Command, Nanjing, China; d Department of Oncology, The First Affiliated Hospital of Nanjing Medical University, Nanjing, China.

**Keywords:** cancer, nomogram, prognosis, survival, terminal stage

## Abstract

Factors influencing the prognosis of patients with terminal-stage cancer remain poorly understood. In this study, we examined these factors and developed a visual model to predict patient survival. Data were collected from patients with terminal-stage cancer treated at the Air Force Hospital of the People’s Liberation Army Eastern Theater Command between 2011 and 2020 were collected. Patients were categorized into the training and validation cohorts. Clinical and laboratory characteristics were collected for analysis and prognostic factors were identified to construct a predictive model, develop a nomogram in the training set (n = 193) and verify it in the validation set (n = 85). Our findings revealed that survival predictions for terminal-stage cancer were not associated with common factors such as tumor type, stage, patient age at diagnosis, or Eastern Cooperative Oncology Group performance status score. Instead, factors such as willingness to receive treatment, dyspnea, serum urea, serum albumin, and neutrophil count proved to be critical. These factors were used to create a highly accurate and reliable nomogram. A comprehensive analysis of prognostic factors in patients with terminal-stage cancer resulted in the development of a practical nomogram model for clinical application.

## 1. Introduction

Early diagnosis of cancer is challenging because of the lack of specific symptoms, resulting in advanced-stage diagnosis.^[1]^ As cancer progresses, conventional therapies become ineffective or intolerable; therefore, palliative and hospice care emerge as primary options during the terminal stage.^[2,3]^ However, there is currently no clear consensus on how to define the terminal stage, as various studies and organizations employed different criteria based on the expected survival time of patients with cancer.^[4,5]^ Consequently, the terminal stages of cancer remain poorly understood.

Patients in the terminal stage approach the end of their lives, and the remaining limited time is extremely precious. If patients and their families could know approximately how long they had left, it would facilitate their arrangement of daily life and work, as well as the formulation of relevant plans. Shifting the treatment goal from curing the disease to alleviating the patient’s pain and improving their quality of life would enable medical staff to formulate better treatment and care plans.

Numerous studies have attempted to identify effective prognostic factors for patients with terminal-stage cancer, including functional status, symptoms, signs, and laboratory parameters.^[6,7]^ Various survival prediction models, such as the palliative performance scale, palliative prognostic score, delirium-palliative prognostic score, palliative prognostic index, and objective prognostic score, have been established to determine these factors.^[8–12]^ In China, palliative and hospice care centers were established relatively late, and research in this field remains scarce.^[13]^ Several prediction tools have been developed by domestic scholars, including the cancer prognosis score, the Chinese prognosis scale, the prognostic scale of survival, and the objective palliative prognosis score, each with its advantages and disadvantages. Compared with the previous models, these models include new prognostic factors and investigate the sensitivity and specificity of scoring tools in different treatment environments. This has facilitated extensive clinical applications.

However, all the existing models are similar. Each prognostic factor included in the scoring tool is assigned a separate value. The total score is then calculated to predict the probability of the patient surviving for several days, weeks, or months. This is inconvenient and fails to intuitively reflect the contribution of each prognostic factor to the prediction.

To address these limitations, nomograms, a type of visual survival model, have gradually applied in cancer research. Scaled line segments were used to represent the impact of each prognostic factor included in the prediction model, simplifying clinical usage and facilitating physician-patient communication. However, there have been few reports on survival prediction in patients in the terminal stage. Our study includes additional prognostic factors and establishes a survival model in the form of a nomogram, making it more widely applicable, intuitive and convenient for clinical use.

## 2. Materials and methods

### 2.1. Patients

This retrospective study involved terminally ill patients with advanced cancer hospitalized at the Air Force Hospital of the People’s Liberation Army Eastern Theater Command between January 1, 2011, and October 31, 2020. The inclusion criteria were as follows: diagnosis of recurrent or metastatic malignant tumors with stage IV; receiving palliative or end-of-life care after available treatment options were exhausted; age ≥ 18 years; routine blood, biochemical, and coagulation function test results within 3 days before or after admission; consciousness at the time of admission; and Eastern Cooperative Oncology Group performance status (ECOG-PS) of 3 to 4; and clinically predicted to survive less than 3 months. The exclusion criteria included patients with 2 or more primary malignant tumors; patients undergoing anti-tumor treatment to actively prolong life; patients experiencing acute or chronic infection within 4 weeks before admission; patients with laboratory parameters potentially interfered with hematological malignancies, autoimmune diseases, or kidney or liver dysfunction; patients on medications, such as hormones, antibiotics, immunosuppressants, coagulation-promoting or anticoagulant drugs. This study was approved by the ethics committee of Air Force Hospital of the People’s Liberation Army Eastern Theater Command and conducted in accordance with the principles of the Declaration of Helsinki.

### 2.2. Study methods

We collected each patient’s demographic data, medical history, clinicopathological data at initial diagnosis, and subsequent anti-tumor treatment history. Moreover, we collected data on symptoms, signs, and laboratory indicators (routine blood, biochemical, and coagulation function) at admission. Based on laboratory data, we calculated the neutrophil-to-lymphocyte ratio, platelet-to-lymphocyte ratio, lymphocyte-to-monocyte ratio, aspartate aminotransferase to alanine aminotransferase ratio, and albumin-to-bilirubin ratio. Previous studies have found that these inflammatory markers are related to cancer prognosis. However, they were not included in the end-stage cancer scoring tools. These clinical and laboratory characteristics were collected for analysis, used to construct a predictive model and develop a nomogram in the training set, and then validated in the validation set. We defined total survival time as the time from admission to death. We followed up all cases for 1 year from the joining date, unless the patient died. The follow-up duration was 100%.

### 2.3. Statistical analysis

All statistical analyses were conducted using R 4.0.3 software. First, we screened each variable based on the missing-value distribution, removing those with more than 20% missing values. To avoid the influence of correlated features, we analyzed the correlations between numerical variables, retaining only a subset based on clinical significance and univariate Cox regression HR values.

Next, we prepared the data by randomly splitting them into training and validation sets in a 7:3 ratio. Continuous variables were converted into binary categorical variables based on the median values in the training set and transformed into binary variables according to clinical practice. We used the chi-square test to compare differences in clinical and pathological factors between the sets.

We excluded dichotomous variables with large deviations from further analysis and performed an initial screening of prognostic factors using univariate survival analysis in 70% of the training set cases. Considering the limit of the total sample size, we retained factors with a *P* value < .1 as significant predictors in the univariate Cox regression analysis.

We used LASSO regression instead of traditional multivariate Cox regression for further feature selection. We selected significant features with non-zero weight coefficients and computed their selection frequency over 200 repetitions. We incorporated the 10 most important factors into multivariate Cox regression models, calculated the C index, corrected the C index for each model, and selected the final model with few features and high discrimination. We visualized the final model as a nomogram and used calibration plots and receiver operating characteristic curves to evaluate its performance in the training and validation sets.

## 3. Results

### 3.1. Patient characteristics

**Figure 1.**
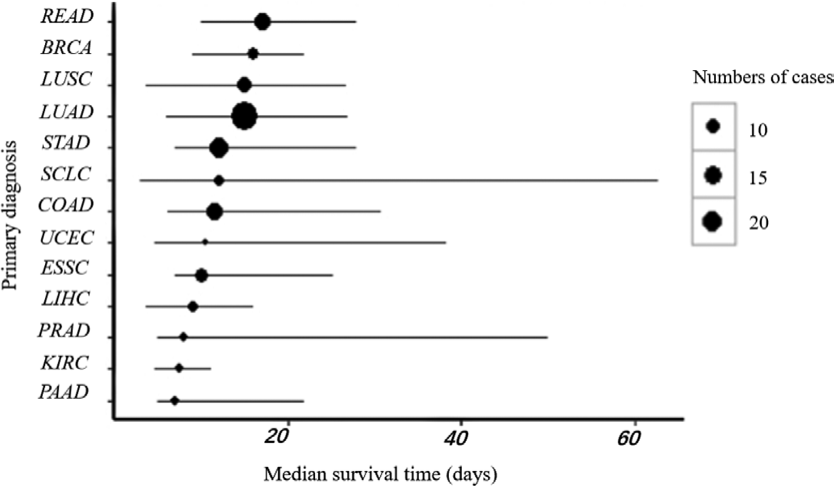
Median survival time of different cancer types in the whole set. BRCA = breast invasive carcinoma, COAD = colon adenocarcinoma, ESSC = esophageal sarcoma, KIRC = kidney renal clear cell carcinoma, LIHC = liver hepatocellular carcinoma, LUAD = lung adenocarcinoma, LUSC = lung squamous cell carcinoma, PAAD = pancreatic adenocarcinoma, PRAD = prostate adenocarcinoma, READ = rectum adenocarcinoma, SCLC = small cell lung cancer, STAD = stomach adenocarcinoma, UCEC = uterine corpus endometrial carcinoma.

This study included 278 patients with terminal cancer (170 males and 108 females). The median age at enrollment was 68 years, and the median survival time was 13 days. Among them, 91 patients had an ECOG-PS score of 3, while 187 had a score of 4. Figure 1 shows the cancer types with at least 10 cases and their corresponding median survival times. Lung adenocarcinoma was the most common type with 63 cases, and a median survival time of 15 days. Gastric adenocarcinoma was the second most common type, with 33 cases and a median survival time of 12 days. Patients with rectal adenocarcinoma had the longest median survival time (17 days).

Owing to the limited sample size, we excluded data with large distribution deviations if the distribution of a dichotomous variable accounted for ≤10% or ≥90% of all samples. We randomly classified the cases into training (193 cases) and validation (85 cases) sets in a 7:3 ratio. The distribution of clinical characteristics in both groups was similar to that of the overall sample. Tables 1 and 2 summarize the clinical data and laboratory indices of the 2 groups. The chi-square test revealed no significant differences in the distribution of characteristics between the groups.

**Table 1:** Clinical features of the training set and validation set.

	The training set	The validation set	*P*		The training set	The validation set	*P*		The training set	The validation set	P
Age				Age at diagnosis				Multiple metastasis			
<68 yr	92 (47.7%)	46 (54.1%)	.389	<66 yr	94 (48.7%)	45 (52.9%)	.603	Yes	98 (50.8%)	35 (41.2%)	.178
≥68 yr	101 (52.3%)	39 (45.9%)		≥66 yr	99 (51.3%)	40 (47.1%)		No	95 (49.2%)	50 (58.8%)	
Sex				Metastasis at diagnosis				Liver metastasis			
Male	74 (38.3%)	34 (40.0%)	.898	0	106 (55.8%)	40 (47.6%)	.263	Yes	74 (38.3%)	32 (37.6%)	1.000
Female	119 (61.7%)	51 (60.0%)		1	84 (44.2%)	44 (52.4%)		No	119 (61.7%)	53 (62.4%)	
Survival status				ECOG-PS				Bone metastasis			
Alive	2 (1.04%)	2 (2.35%)	.588	3	62 (32.1%)	29 (34.1%)	.851	Yes	67 (34.7%)	26 (30.6%)	.593
Dead	191 (99.0%)	83 (97.6%)		4	131 (67.9%)	56 (65.9%)		No	126 (65.3%)	59 (69.4%)	
Tobacco				Temperature				Lung metastasis			
Yes	31 (16.1%)	12 (14.1%)	.803	<36.5°C	75 (38.9%)	33 (38.8%)	1.000	Yes	88 (45.6%)	34 (40.0%)	.462
No	161 (83.9%)	73 (85.9%)		≥36.5°C	118 (61.1%)	52 (61.2%)		No	105 (54.4%)	51 (60.0%)	
Hepatitis				Pulse or heart rate				Brain metastasis			
Yes	31 (16.1%)	10 (11.8%)	.455	<81/min	95 (49.2%)	47 (55.3%)	.422	Yes	42 (21.8%)	13 (15.3%)	.593
No	162 (83.9%)	75 (88.2%)		≥81/min	98 (50.8%)	38 (44.7%)		No	151 (78.2%)	72 (84.7%)	
Operation history				Respiratory rate				Pathological stage at diagnosis			
Yes	88 (46.1%)	35 (41.7%)	.586	<18/min	68 (35.2%)	32 (37.6%)	.802	I–III	67 (43.8%)	30 (40.5%)	.748
No	103 (53.9%)	49 (58.3%)		≥18/min	125 (64.8%)	53 (62.4%)		IV	86 (56.2%)	44 (59.5%)	
Chemotherapy				Systolic pressure				Weight loss			
Yes	108 (58.1%)	51 (61.4%)	.699	<124 mm Hg	32 (37.6%)	53 (62.4%)	.070	≤3 kg/2 wk	154 (79.8%)	68 (85.0%)	.484
No	78 (41.9%)	32 (38.6%)		≥124 mm Hg	53 (62.4%)	32 (37.6%)		<3 kg/2 wk	19 (11.0%)	12 (15.0%)	
Intake				Diastolic pressure				Pleural diffusion			
Normal	67 (34.7%)	29 (34.1%)	1.000	<75 mm Hg	96 (49.7%)	43 (50.6%)	.811	Yes	18 (9.42%)	11 (13.1%)	.484
Decrease	126 (65.3%)	56 (65.9%)		≥75 mm Hg	97 (50.3%)	42 (49.4%)		No	173 (90.6%)	73 (86.9%)	
Willingness of therapy				Visual analogue scale				Pleural diffusion			
Negative	143 (74.1%)	60 (70.6%)	.646	≤3	105 (54.4%)	46 (54.1%)	1.000	Yes	18 (9.42%)	11 (13.1%)	.484
Positive	50 (25.9%)	25 (29.4%)		>3	88 (45.6%)	39 (45.9%)		No	173 (90.6%)	73 (86.9%)	
Analgesic or sedative drugs use				Anorexia				Pain			
Yes	92 (47.7%)	42 (49.4%)	.890	Yes	37 (19.5%)	10 (11.8%)	.163	Yes	108 (56.0%)	49 (57.6%)	.896
No	101 (52.3%)	43 (50.6%)		No	153 (80.5%)	75 (88.2%)		No	85 (44.0%)	36 (42.4%)	
Glucocorticoid use				Nausea				Abdominal pain			
Yes	37 (19.2%)	15 (17.6%)	.894	Yes	39 (20.4%)	24 (28.2%)	.203	Yes	28 (14.5%)	20 (23.5%)	.097
No	156 (80.8%)	70 (82.4%)		No	152 (79.6%)	61 (71.8%)		No	165 (85.5%)	65 (76.5%)	
Furosemide use				Vomiting				Bone pain			
Yes	39 (20.2%)	13 (15.3%)	.423	Yes	34 (17.8%)	18 (21.2%)	.620	Yes	24 (12.6%)	4 (4.71%)	.073
No	154 (79.8%)	72 (84.7%)		No	157 (82.2%)	67 (78.8%)		No	166 (87.4%)	81 (95.3%)	
Expiratory dyspnea				Astriction				Pantalgia			
Yes	61 (31.6%)	25 (29.4%)	.823	Yes	28 (14.7%)	13 (15.3%)	1.000	Yes	22 (11.4%)	9 (10.6%)	1.000
No	132 (68.4%)	60 (70.6%)		No	63 (85.3%)	72 (84.7%)		No	171 (88.6%)	76 (89.4%)	
Neuromotor dysfunction of limbs				Insomnia				Pleural effusion			
Yes	24 (12.6%)	6 (7.06%)	.251	Yes	70 (36.6%)	37 (43.5%)	.342	Yes	22 (11.4%)	11 (12.9%)	.869
No	167 (87.4%)	79 (92.9%)		No	121 (63.4%)	48 (56.5%)		No	171 (88.6%)	74 (87.1%)	
Site of primary cancer				Cough				Ascites			
Respiratory	65 (33.7%)	29 (34.1%)	.644	Yes	85 (44.5%)	43 (50.6%)	.421	Yes	19 (9.84%)	13 (15.3%)	.268
Digestive	85 (40.0%)	42 (49.4%)		No	106 (55.5%)	42 (49.4%)		No	174 (90.2%)	72 (84.7%)	
Urogenital	36 (18.7%)	13 (15.3%)									
Others	7 (3.6%)	1 (1.2%)									
Appetite loss				Taste change				Dropsy			
Yes	118 (61.8%)	56 (65.9%)	.605	Yes	33 (17.3%)	20 (23.5%)	.293	Yes	42 (21.8%)	22 (25.9%)	.550
No	73 (38.2%)	29 (34.1%)		No	158 (82.7%)	65 (76.5%)		No	151 (78.2%)	63 (74.1%)	
Fatigue				Olfactory change				Distraction			
Yes	124 (64.9%)	55 (64.7%)	1.000	Yes	30 (15.8%)	11 (12.9%)	.667	Yes	22 (11.6%)	16 (18.8%)	.156
No	67 (35.1%)	30 (35.3%)		No	160 (84.2%)	74 (87.1%)		No	168 (88.4%)	69 (81.2%)	

**Table 2:** Laboratory indexes of the training set and validation set.

	The training set	The validation set	*P*		The training set	The validation set	*P*		The training set	The validation set	*P*
WBC (×10^9^/L)				ALT (U/L)				Glu (mmol/L)			
<9.1	91 (49.2%)	41 (49.4%)	1.000	<16.95	94 (50.0%)	36 (43.4%)	.382	<5.49	93 (50.0%)	38 (45.8%)	.612
≥9.1	94 (50.8%)	42 (50.6%)		≥16.95	94 (50.0%)	47 (56.6%)		≥5.49	93 (50.0%)	45 (54.2%)	
Neu (×10^9^/L)				AST(U/L)				Ca (mmol/L)			
<7.2	90 (48.6%)	39 (47.0%)	.905	<28.4	94 (50.0%)	43 (51.8%)	.887	<2.21	92 (48.9%)	37 (44.6%)	.596
≥7.2	95 (51.4%)	44 (53.0%)		≥28.4	94 (50.0%)	40 (48.2%)		≥2.21	96 (51.1%)	46 (55.4%)	
Lym (×10^9^/L)				GGT (U/L)				Na (mmol/L)			
<0.9	91 (49.2%)	38 (45.8%)	.701	<61.2	94 (50.0%)	42 (50.6%)	1.000	<136.7	94 (50.0%)	35 (42.2%)	.290
≥0.9	94 (50.8%)	45 (54.2%)		≥61.2	94 (50.0%)	41 (49.4%)		≥136.7	94 (50.0%)	48 (57.8%)	
Lym%				Tbil (µmol/L)				K (mmol/L)			
<9.7	92 (49.7%)	37 (45.1%)	.574	<12.47	93 (50.0%)	34 (41.5%)	.247	<3.91	94 (50.0%)	47 (56.6%)	.382
≥9.7	93 (50.3%)	45 (54.2%)		≥12.47	93 (50.0%)	48 (58.5%)		≥3.91	94 (50.0%)	36 (43.4%)	
Neu%				Dbil (µmol/L)				Cl (mmol/L)			
<83.1	92 (49.7%)	48 (57.8%)	.273	<5.705	93 (50.0%)	39 (47.0%)	.746	<97.65	94 (50.0%)	37 (44.6%)	.489
≥83.1	93 (50.3%)	35 (42.2%)		≥5.705	93 (50.0%)	44 (53.0%)		≥97.65	94 (50.0%)	46 (55.4%)	
Mon (×10^9^/L)				Ibil (µmol/L)				NLR			
<0.6	89 (48.1%)	41 (49.4%)	.950	<6.74	93 (50.0%)	41 (50.0%)	1.000	<8.5	92 (49.7%)	43 (51.8%)	.855
≥0.6	96 (51.9%)	42 (50.6%)		≥6.74	93 (50.0%)	41 (50.0%)		≥8.5	93 (50.3%)	40 (48.2%)	
Mon%				BUN (mmol/L)				LMR			
<6.5	92 (49.7%)	41 (49.4%)	1.000	<6.49	94 (50.0%)	40 (48.2%)	.887	<1.57	90 (48.6%)	47 (56.6%)	.282
≥6.5	93 (50.3%)	42 (50.6%)		≥6.49	94 (50.0%)	43 (51.8%)		≥1.57	95 (51.4%)	36 (43.4%)	
RBC (×10^12^/L)				Cr (µmol/L)				PLR			
<3.33	92 (49.7%)	37 (44.6%)	.517	<64.0	93 (49.7%)	38 (45.8%)	.640	<207.86	92 (49.7%)	49 (59.0%)	.201
≥3.33	93 (50.3%)	46 (55.4%)		≥64.0	94 (50.3%)	45 (54.2%)		≥207.86	93 (50.3%)	34 (41.0%)	
Hb (g/L)				UA (µmol/L)				ABR			
<97	89 (48.1%)	35 (42.2%)	.442	<282.2	93 (49.7%)	37 (45.1%)	.573	<2.26	92 (49.7%)	42 (51.2%)	.927
>97	96 (51.9%)	48 (57.8%)		≥282.2	94 (50.3%)	45 (54.9%)		≥2.26	93 (50.3%)	40 (48.8%)	
PLT (×10^9^/L)				ALB (g/L)				SLR			
<183	91 (49.2%)	51 (61.4%)	.084	<29.2	92 (49.2%)	38 (45.8%)	.699	<1.82	94 (50.0%)	49 (59.0%)	.214
≥183	94 (50.8%)	32 (38.6%)		≥29.2	95 (50.8%)	45 (54.2%)		≥1.82	94 (50.0%)	41.0%)	

ABR = albumin-to-bilirubin ratio, ALB = albumin, ALT = alanine aminotransferase, AST = aspartate aminotransferase, BUN = blood urea nitrogen, Ca = calcium, Cl = chloride, Cr = creatinine, Dbil = direct bilirubin, GGT = gamma-glutamyl transferase, Glu = glucose, Hb = hemoglobin, K = potassium, LMR = lymphocyte-to-monocyte ratio, Lym = lymphocyte count, Mon% = monocyte percentage, Na = sodium, Neu = neutrophil count, NLR = neutrophil-to-lymphocyte ratio, PLR = platelet-to-lymphocyte ratio, PLT = platelet count, RBC = red blood cell count, SLR = aspartate aminotransferase to alanine aminotransferase ratio, UA = uric acid, WBC = white blood cell count.

### 3.2. Analysis of prognostic factors

**Figure 2.**
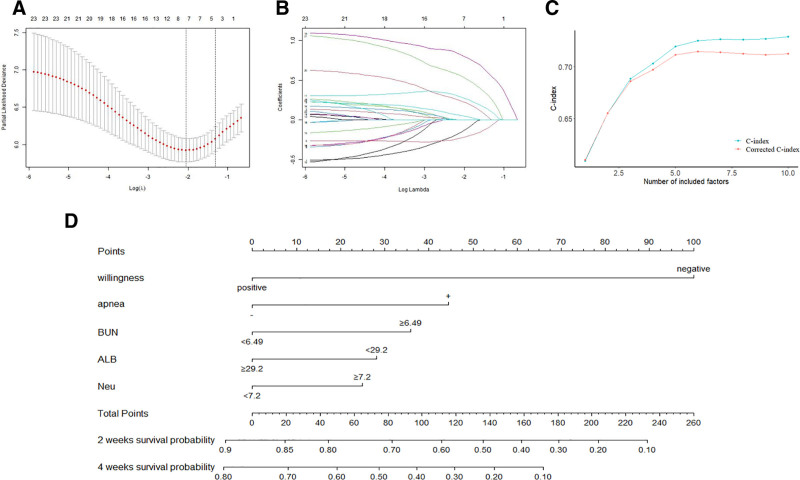
Selection of prediction using Lasso regression analysis in the training set (A). Using 10-fold cross-validation, the dotted vertical lines were drawn at the optimal values by the minimum criteria and 1-s.e. criteria (B). At the optimal value by the minimum criteria, 7 variables were selected. The C-index and the corrected C-index of the model are shown with different features (C). Along with the nomogram predicting 2-wk and 4-wk survival based on the training set (D). ALB = albumin, BUN = blood urea nitrogen, Neu = neutrophil count.

We randomly selected 70% of the data from the training set using the sample function in R. Univariate Cox survival analysis was performed to screen prognostic factors. As presented in Table 3, the independent prognostic factors for terminal-stage cancer included age, history of chemotherapy, weight loss, general pain, edema, insomnia, resting dyspnea, peripheral motor nerve disorders, olfactory changes, ECOG-PS, nutritional intake, willingness to treat, brain metastasis, pulse or heart rate, respiratory rate, systolic blood pressure, neutrophil count (Neu), monocyte percentage, hemoglobin, albumin (ALB), aspartate aminotransferase, direct bilirubin and blood urea nitrogen (BUN). Subsequently, we performed further feature selection using LASSO regression, as illustrated in Figure 2A and B. We selected the total frequency corresponding to the different characteristics by randomly sampling 200 times. The most important characteristics (top 10 frequencies, from high to low) included willingness to treat, dyspnea, BUN, ALB, Neu, hemoglobin, nutritional intake, insomnia, respiratory frequency, and monocyte percentage.

**Table 3:** Univariate Cox survival analysis.

Univariate Cox survival analysis				Univariate Cox survival analysis				Univariate Cox survival analysis			
	HR	95% CI	*P*		HR	95% CI	*P*		HR	95% CI	*P*
Sex	1.137	(0.790–1.635)	.489	Fatigue	0.951	(0.665–1.361)	.785	Analgesic or sedative drugs use	1.114	(0.789–1.572)	.539
Age at diagnosis	1.463	(1.035–2.068)	.031*	Expiratory dyspnea	2.530	(1.704–3.755)	.000*	Glucocorticoid use	0.745	(0.480–1.154)	.188
Tobacco	0.707	(0.446–1.120)	.139	Anorexia	1.048	(0.666–1.650)	.839	Furosemide use	1.389	(0.898–2.147)	.140
Hepatitis	0.885	(0.575–1.363)	.579	Astriction	0.774	(0.484–1.238)	.285	Temperature	1.127	(0.792–1.604)	.506
Respiratory	1.060	(0.450–2.500)	.894	Nausea	0.808	(0.535–1.220)	.310	Pulse or heart rate	1.963	(1.358–2.837)	.000*
Digestive	0.169	(0.509–2.759)	.695	Vomiting	0.813	(0.558–1.183)	.376	Respiratory rate	1.511	(1.050–2174)	.026*
Urogenital	0.182	(0.495–2.908)	.687	Taste change	0.988	(0.621–1.569)	.958	Systolic pressure	1.341	(0.949–1.894	.096*
Other cancers	R	–	–	Appetite loss	0.904	(0.632–1.293)	.580	Neu	1.901	(1.331–2.715)	.000*
Metastasis at diagnosis	0.984	(0.693–1.399)	.930	Neuromotor dysfunction of limbs	0.545	(0.307–0.969)	.039*	Mon%	0.704	(0.496–1.000)	.050*
Pathological stage at diagnosis	1.017	(0.694–1.488)	.932	Distraction	1.314	(0.787–2.194)	.297	PLT	0.967	(0.682–1.370)	.849
Operation history	1.170	(0.825–1.658)	.379	Olfactory change	0.485	(0.293–0.805)	.005*	Hb	0.587	(0.407–0.848)	.004*
Chemotherapy	0.627	(0.440–0.893)	.010*	Cough	1.091	(0.770–1.545)	.624	ALB	0.431	(0.298–0.624)	.000*
Visual analogue scale	1.023	(0.725–1.443)	.897	ECOG-PS	1.892	(1.301–2.753)	.001*	AST	1.661	(1.155–2.388)	.006*
Weight loss	2.319	(1.155–4.658)	.018*	Intake	2.361	(1.618–3.444)	.000*	Glu	1.129	(0.793–1.607)	.501
Pain	0.995	(0.703–1.410)	.979	Willingness of therapy	3.974	(2.392–6.603)	.000*	Dbil	1.475	(1.036–2.098)	.031*
Abdominal pain	1.187	(0.734–1.921)	.484	Multiple metastasis	0.748	(0.528–1.058)	.101	BUN	2.396	(1.672–3.434)	.000*
Bone pain	0.910	(0.538–1.539)	.724	Liver metastasis	1.348	(0.938–1.939)	.107	Ca	0.790	(0.557–1.119)	.185
Pantalgia	1.817	(1.063–3.106)	.029*	Bone metastasis	0.978	(0.686–1.396)	.904	LMR	0.823	(0.579–1.169)	.277
Pleural effusion	1.424	(0.851–2.382)	.178	Lung metastasis	0.990	(0.702–1.395)	.954	SLR	1.288	(0.909–1.824)	.154
Ascites	1.399	(0.800–2.446)	.239	Brain metastasis	0.576	(0.379–0.874)	.010*				
Dropsy	1.486	(0.949–2.328)	.084*	Pleural diffusion	1.435	(0.747–2.755)	.278				

Ca = calcium, Glu = glucose, PLT = platelet count.

* *P* < .1.

### 3.3. Prognosis model and nomogram construction

We sequentially incorporated the top 10 features into the multivariate Cox regression model, calculated the C-index of each model, and corrected it after 10-fold cross-validation. Figure 2C shows a line chart of the corresponding model. The C-index of the model exceeded 0.70 after including 5 features, indicating a better degree of differentiation. Conversely, adding more prognostic factors did not improve model’ differentiation and increased the risk of reduced convenience and reliability. Therefore, we selected 5 prognostic factors treatment willingness, dyspnea, BUN, ALB and Neu to establish a survival prognosis model. The results revealed that patients’ willingness to treat had the most significant impact on prognosis, followed by dyspnea, BUN, ALB, and Neu. Because the cases involved terminal-stage patients with poor physical conditions, clinicians predicted a survival time of 3 months. The median survival time was approximately 2 weeks, and the total survival time was ≤4 weeks. We set the predicted survival time points to 2 and 4 weeks. Based on the training set, the models were visualized as nomograms (Fig. 2D).

### 3.4. Model validation

**Figure 3.**
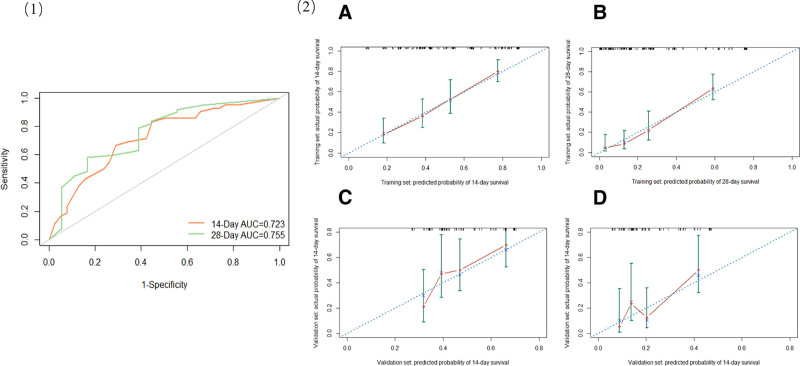
ROC curves for the validation set at 14 and 28th day (1); Calibration curves of the nomogram for 14-d (2A) and 28-d (2B) survival in the training set; calibration curves for 14-d (2C) and 28-d (2D) survival in the validation set. AUC = area under the curve, ROC = receiver operating characteristic.

We used the results from the 85 cases in the validation set to confirm the survival prognosis model. The receiver operating characteristic curve indicated that the area under the curve values on the14th and 28th days of the validation set exceeded 0.7, demonstrating a good discrimination (Fig. 3(1)). These findings collectively revealed that the model’s predicted survival rates on the 14th and 28th days were generally consistent with the actual survival rates, thus establishing the high reliability of our prognostic model (Fig. 3 (2)).

## 4. Discussion

The prediction of survival time exceeding 3 months for patients with terminal stage cancer has been closely associated with tumor type, stage, age, and ECOG-PS score in numerous clinical studies. However, patients with a predicted survival time of ≤3 months are often excluded from most cancer clinical trials, leading to limited reports and no consensus on their survival prediction. With few existing survival models for patients with terminal-stage cancer in China, where palliative and hospice care are relatively recent developments, this study aimed to predict patient survival times to provide practical evidence for clinicians.

Our findings revealed that the common prognostic factors for non-terminal-stage cancers, such as tumor type, initial tumor stage, age, and ECOG-PS score, were not statistically relevant to the prognosis of patients with terminal-stage cancer with an expected survival time of ≤3 months. This association may arise from differences in treatment plans based on tumor stage, age, and ECOG-PS scores during the early stages of cancer, leading to variations in prognosis. In the terminal stage, patients usually undergo multiple treatment courses, experience cancer metastases, have a high cancer burden, and have a poor overall condition. Consequently, differences in body function reflected by tumor type, age and ECOG-PS scores may have a limited impact on prognosis. Notably, our study identified critical prognostic factors, including patient’ willingness to undergo treatment, dyspnea symptoms, serum ALB levels, BUN levels, and Neu. Among these, willingness to treat emerged as a significant positive factor for prognosis after LASSO regression screening. Positive attitudes can help patients make the most of their remaining time and find meaning and fulfillment even at the terminal stage.^[14]^ Our findings align with Barbot’s study,^[15]^ which revealed the connection between survival time and patient’ treatment attitudes in terminal cancer cases. A negative attitude corresponded to a 22-day median survival, whereas moderate and positive attitudes led to 54- and 126-day survival, respectively. Negative attitudes led to shorter survival, whereas positive attitudes improved the prognosis. This highlights the importance of focusing on the patients’ mental states and exploring appropriate interventions.

Dyspnea, a prevalent clinical symptom,^[16,17]^ is a crucial prognostic factor in patients with terminal-stage cancer. In this study, dyspnea incidence (31.6%) was consistent with those of previous findings,^[18–20]^ and was determined to be major prognostic factor via collinear analysis and LASSO regression screening. The etiology of dyspnea was similar to that reported in other studies, including pulmonary tumors and pleural effusions.^[21]^

BUN, a byproduct of protein metabolism, varies owing to factors such as a high-protein diet, hormone usage, and specific diseases.^[22,23]^ We found that higher BUN levels indicated a worse prognosis in patients with terminal cancer, possibly because of increased tumor consumption, protein catabolism, and hypoproteinemia. Malnutrition and excessive protein intake can also cause BUN levels to surpass the metabolic capacity of the body. Although BUN is linked to kidney disease and heart failure prognoses,^[24,25]^ only a few studies have explored its relationship with cancer prognosis.^[26]^ This index could serve as a potential laboratory indicator for terminal-stage cancer prognosis. Increased BUN levels may also indicate decreased serum ALB levels, suggesting a poor prognosis.

Malnutrition, often indicated by low serum ALB levels, is common in patients with terminal-stage cancer and is correlated with shorter survival times.^[27]^ Decreased immunity due to malnutrition increases the risk of pulmonary infections and thrombosis, leading to a worse prognosis.^[28,29]^ Our study supports the finding that lower serum ALB levels and increased Neu are independent prognostic factors in these patients.

Nomograms have recently gained popularity because they graphically represent the effects of prognostic factors on survival.^[30–33]^ However, their use in terminal-stage cancer evaluation is limited. We developed a multivariate Cox regression model, which was visualized as a nomogram, using data from 278 patients. This model considers factors such as patients’ willingness to receive treatment, dyspnea, and serum BUN, ALB, and Neu to calculate survival rates. Eighty-five additional cases were assessed to ensure the reliability of the model. The predicted 14 and 28-day survival rates were consistent with the actual rates, demonstrating the reliability of the model. Our study provides a practical and effective prognostic model for predicting patient survival.

Nonetheless, our study had some limitations. First, this was a single-center study with a relatively small sample size. This study was limited by the characteristics of the patient population at specific medical institutions. Hence, this can limit the generalizability of our results to all central populations because it is unknown whether there are regional or racial differences. Second, this was a retrospective study, some information was missing. Incomplete data from some areas may have led to a bias. Therefore, prospective, large-scale studies are required to address these limitations. To ensure the reliability and reproducibility of our findings, we employed a continuous patient recruitment process, maintained a low rate of loss at follow-up, and collected comprehensive data. In addition, we used various reliable statistical methods to produce robust and replicable results, contributing to the scientific rigor of our investigation.

## Author contributions

**Conceptualization:** Lingxiang Liu.

**Data curation:** Weiwei Gui, Lei Xu.

**Formal analysis:** Weiwei Gui.

**Writing – original draft:** Weiwei Gui.

**Writing – review & editing:** Lingxiang Liu, Weiwei Gui, Chunhui Ding, Lei Xu, Yizhou Luo.
